# Is *Fusobacterium nucleatum* the key mediator between oral infections and systemic diseases? Mechanistic insights and therapeutic implications

**DOI:** 10.1080/19490976.2026.2694819

**Published:** 2026-06-30

**Authors:** Gautham Siddarth Ragunagam, Anand Anbarasu, Gothandam Kodiveri Muthukaliannan

**Affiliations:** a Department of Biotechnology, School of Bio Sciences and Technology, Vellore Institute of Technology, Vellore, Tamil Nadu, India; b Medical and Biological Computing Laboratory, School of Bio Sciences and Technology, Vellore Institute of Technology, Vellore, Tamil Nadu, India

**Keywords:** *Fusobacterium nucleatum*, oral dysbiosis, microbial translocation, systemic diseases, host–microbiome interactions

## Abstract

*Fusobacterium nucleatum* has emerged as a pathobiont that associates oral dysbiosis with systemic diseases through coaggregation, hematogenous dissemination, and immune modulation. This review provides molecular insights through which they are involved in systemic diseases such as colorectal cancer, adverse pregnancy outcomes, cardiovascular diseases, neurodegenerative disorders, and diabetes mellitus. Key virulence factors include the adhesins of FadA, Fap2, and RadD, lipopolysaccharides, and outer membrane vesicles, which mediate epithelial invasion and endothelial permeafbility and facilitate immune suppression through TLR4-NF-κB, β-catenin/Wnt, and MAPK signaling pathways. These interactions result in impaired tissue homeostasis, propagate chronic inflammation, and promote oncogenic and metabolic modulation. Systemic pleiotropy of *F. nucleatum* is further substantiated by its involvement in chemoresistance, placental dysfunction, vascular inflammation, and neuronal injury, substantiating its systemic pleiotropy. Emerging therapeutic strategies, such as blocking adhesins, neutralizing outer membrane vesicles, microbiome manipulation, and using CRISPR-based clearance, provide precision techniques for mitigating diseases. Therefore, this review identifies *F. nucleatum* as the primary microbial mediator of oral-systemic pathology and its translational significance in the development of targeted antimicrobial and host-directed therapies.

## Introduction

The aggregation or co-aggregation of microorganisms on biotic or abiotic surfaces forms biofilms. The human oral cavity renders a highly active ecosystem for microbes inhabited by various bacterial species competing for niche colonization and biofilm formation.^1^ The oral ecosystem consists of various microorganisms, including initial and late colony formers, which form biofilms called dental plaques. *Streptococcus oralis, Streptococcus gordonii, Streptococcus sanguinis, and Streptococcus salivarius* are the most prevalent native initial colonizers in the human oral cavity that secrete antimicrobial peptides and small molecules when they develop dental plaque or biofilms.^2^ The late transient colonizers, such as *Escherichia coli*, *Staphylococcus aureus*, *Streptococcus pneumoniae*, *Haemophilus influenzae*, *Acinetobacter baumanii*, and *Pseudomonas aeruginosa,* colonize the human oral environment through food, beverages, or interpersonal contact and play a crucial role in modulating the microbial homeostasis of the human oral environment.^2^

The most common bacteria found in the oral cavity of humans are *Fusobacterium nucleatum*. They are characterized by their extensive coaggregation abilities with a wide variety of microbial species interactions.^3^ They are gram-negative, spindle-shaped, oral anaerobic microorganisms that play a crucial role as a bridging organism in microbial community dynamics because they are positioned between early and late colonizers.^4^ Genomically, they exhibit a large number of adhesin proteins, outer membrane proteins, and virulence factors that make them ecologically successful. Moreover, their adaptability at a metabolic level and production of Short-Chain Fatty Acids (SCFA) generate a pro-inflammatory microenvironment that favors the shift between microbial homeostasis and dysbiosis. These characteristics suggest that they are not only a potential biofilm inhabitant but also active as a microbe that can induce both local and systemic pathology.^5^

*F. nucleatum* is a key stakeholder in the onset and development of periodontal diseases, including gingivitis and periodontitis. Their coaggregation capacity is responsible for the structural maturation of the subgingival biofilm by connecting early commensals and late pathogens, contributing to the enhanced microbial diversity and community stability.^6^ *F. nucleatum* infects the gingival epithelium via adhesion and invading epithelial cells with the help of the Fusobacterium adhesin A (FadA) adhesin, which is a key virulence factor involved in host‒pathogen interactions. These events alter epithelial barrier function and lead to the activation of pro-inflammatory signaling cascades, such as the nuclear factor kappa B (NF-κB) and mitogen-activated protein kinase (MAPK) pathways, resulting in increased production of cytokines such as interleukin-1beta (IL-1β), interleukin-6 (IL-6), interleukin-8 (IL-8), and tumor necrosis factor-alpha (TNF-α). These cytokines enhance the local immune response, connective tissue degradation, and alveolar bone loss.^7^ Interaction with other periodontal pathogens, notably *Porphyromonas gingivalis*, displays synergistic pathogenicity by increasing biofilm virulence and immune resistance mechanisms, including protease-mediated complement inactivation.^8^ These multidimensional functions indicate that *F. nucleatum* is not only a secondary colonizer but also a functionally and pathologically important participant in chronic inflammatory conditions in periodontitis.

*F. nucleatum* has been identified as an eminent oral microbial pathogen linked to a rapidly growing number of systemic conditions. They can able to translocate from the oral cavity into the bloodstream and colonize remote tissues, where they participate in chronic inflammation, immune modulation, and histology. In colorectal cancer (CRC), they promote the growth and metastasis of tumor cells with FadA-mediated activation of the Wnt/β-catenin pathway and Fusobacterium autotransporter protein 2 (Fap2)-mediated adhesion to D-galactose-β(1-3)-N-acetyl-D-galactosamine (Gal-GalNAc)-expressing tumor cells to suppress host anti-tumor immunity interactions with T cell immunoglobulin and ITIM domain (TIGIT) and carcinoembryonic antigen-related adhesion molecule 1 (CAECAM1).^9^ Detection of *F. nucleatum* in atherosclerotic plaques suggests their contribution to vascular endothelial dysfunction and plaque destabilization is induced by the upregulation of pro-inflammatory cytokines and the activation of the immunological response, in terms of increasing cardiovascular diseases.^10^ *In vitro* analyses have demonstrated that *F. nucleatum* colonizes placental and amniotic tissues, causing fetal membrane degradation through Toll-like receptor (TLR)-induced inflammation, suggesting its involvement in gestational disorders such as preterm delivery and stillbirth.^11^ The existence of proinflammatory metabolites of this bacterium in brain tissues suggests an important role in the induction of neuroinflammatory diseases and neuronal injury.^12^ These various pathological associations highlight the ability to invade mucosal barriers, escape immune surveillance, and trigger an inflammatory response at remote sites. These findings consider *F. nucleatum* as a pleomorphic microbial etiology of systemic illness, beyond the oral cavity.

### *F. nucleatum*: the biofilm architect of oral biofilm

**Figure 1.**
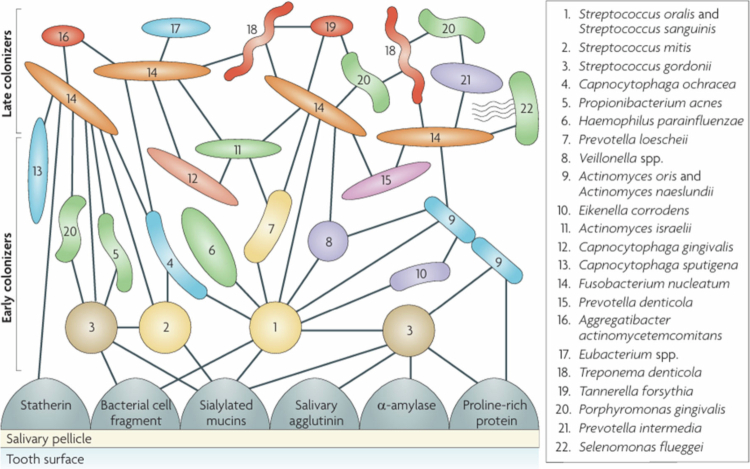
Spatial‒temporal framework of oral bacteria: this figure demonstrates the colonisation pattern of oral microbes in the human oral cavity. Early colonizers, such as *Streptococci*, *Actinomyces*, and *Veillonella*, initially bind to host-derived salivary components, including statherin, sialylated mucins, α-amylase, and proline-rich proteins. *F. nucleatum* acts as a bridging organism, enabling early and late colonizers to adhere to the salivary pellicle, thereby organizing a mature biofilm. Late colonizers, including *P. gingivalis* and *Treponema denticola*, contribute to the complexity of the biofilm (image adapted from ref.^3^).

*F. nucleatum* is a major player in the coordination of the oral biofilm architecture by acting as a structural bridge linking early-colonizing commensals with late-colonizing pathogens (Figure 1).^3^ Their elongated fusiform morphology and ability to coaggregate with a wide range of microbial species make it a physical and metabolic link within the biofilm matrix.^13^ Such spatial integration makes the vertical as well as horizontal organization of biofilms promote microbial succession and community robustness. This microorganism expresses an incredible repertoire of adhesin proteins and outer membrane proteins, such as arginine-inhibitable adhesin (RadD), Fap2, coaggregation mediating protein A (CmpA), fusobacterial outer-membrane protein A (FomA), and adherence-inducing determinant 1 (Aid1), which mediate certain interbacterial interactions.^14^

**Figure 2.**
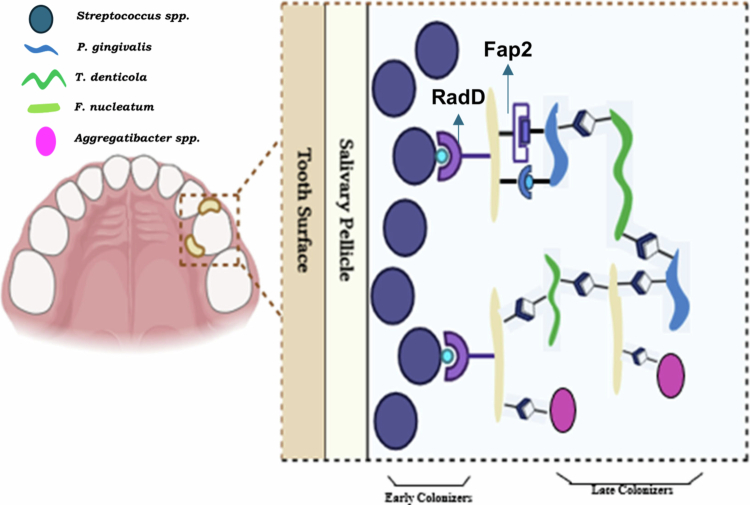
Schematic representation showing the ability of *F. nucleatum* to function as a bridging organism: *F. nucleatum* acts as a key bridging organism connecting early colonizers with late colonizers (periodontal pathogens). This interaction is mediated by multiple adhesins, including RadD, which helps in coaggregation with early colonizers, and Fap2 promotes binding to late colonizers. These adhesin-mediated interactions enable the integration of late colonizers into developing biofilms.

RadD is an important adhesin that mediates coaggregation with several gram-positive bacteria, including *S. sanguinis*, *S. gordonii*, and *Actinomyces naeslundii*, to form the initial binding layer of oral biofilm (Figure 2). RadD participates in a calcium ion-mediated interaction with the surface molecules of *Streptococcus* sp., forming a stable interbacterial complex crucial for the initial development of biofilms.^15^ Fap2, a galactose-inhibitable binding protein, is involved in interactions with gram-negative anaerobes such as *P. gingivalis* and *Prevotella* sp. (Figure 2), but they also display immune-modulatory activity by binding to the human TIGIT receptor present on lymphocytes, which helps in immune evasion in the inflammation sites of periodontitis.^16^ CmpA facilitates high-affinity binding to both Gram-positive and Gram-negative species, promoting lateral spread within the developing biofilm. Aid1 controls the specificity of coaggregation and modulates the binding profile of *F. nucleatum*, especially under pH conditions.^17^

These adhesins act synergistically rather than independently and enable the colonization of *F. nucleatum* as a structural intermediate that promotes vertical stratification and lateral growth of polymicrobial biofilm. The interplay among these surface proteins provides *F. nucleatum* the structural flexibility of the biofilm matrix and thus increased the structural integrity and ecological adaptability of the microbial consortium.^15-17^

### *F. nucleatum*: oral disease immunomodulation and host interaction

*F. nucleatum* not only contributes to the development of oral biofilm formation but also plays an important role in periodontal diseases through direct host‒microbe interactions and the regulation of innate immunity. Their colonization in subgingival plaques and vicinity of the gingival epithelium facilitates their engagement with host receptors, allowing disruption of mucosal barriers and initiating pro-inflammatory signaling.^18^ The FadA adhesin protein is a key virulence factor that binds to epithelial cadherin (E-cadherin) on epithelial cells, which results in disintegration of adherent junctions, increased paracellular permeability, and the translocation of microbes into connective tissue. This interaction also triggers β-catenin signaling pathway, leading to the nuclear translocation of β-catenin and the transcription of genes involved in cell proliferation and pro-survival response.^19^

Simultaneously, the lipopolysaccharides (LPS) and the outer membrane vesicles (OMVs) activate TLR2 and TLR4 receptors and therefore initiate myeloid differentiation primary response 88 (MyD88)-dependent NF-κB and MAPK cascades. These effects result in the production of important pro-inflammatory cytokines, such as IL-1β, IL-6, IL-8, and TNF-α, which mediate the recruitment and activation of neutrophils and macrophages, thereby leading to local inflammatory responses and contributing to damage of periodontal tissue.^20^ OMV-associated components, such as proteases and cytotoxic metabolites, mediate the immune system manipulation by degrading immunoglobulins and preventing the complement activation, thereby aiding the immune evasion.^21^ In addition, the synergistic interaction of *F. nucleatum* with other oral pathogens, such as *P. gingivalis*, enhances immune resistance and disruption of tissues. The overall mechanisms highlight *F. nucleatum* as a central immune-modulating pathogen in the pathogenesis of periodontitis.^9^

### *F. nucleatum*: systemic propagation and virulence beyond the oral cavity

*F. nucleatum* is considered one of the most important oral pathogens because they translocate from the oral cavity into distant organ systems. fascinating ability to translocate from the oral cavity into distant organ systems. The primary cause of hematogenous dissemination is attributed to transient bacteremia caused by periodontal inflammation or mechanical disruption of the gingival epithelium.^9^ They use their adhesins, OMVs, and metabolic byproducts in the bloodstream to access the host tissues, manipulate immune responses, and drive persistent infection.^19^ Accumulating evidence incriminates this bacterium in the pathogenesis of a variety of systemic illnesses, with mechanisms that transgress passive microbial translocation to active molecular cross-talk involving host signaling pathways. Systemically, they have been implicated in CRC, pregnancy complications, cardiovascular diseases, and neurodegenerative disorders, replicating their pleiotropic pathogenic potential beyond the oral cavity.^22^

### Colorectal cancer

CRC is the well-established systemic association of *F. nucleatum* with clinical, molecular, and mechanistic evidence.^9^ ^,^ ^14^ ^,^ ^23-25^ Selective enrichment of *F. nucleatum* in colorectal tumor tissues relative to contiguous normal mucosa has been consistently shown by high-resolution 16S rRNA sequencing, shotgun metagenomics, and quantitative polymerase chain reaction (qPCR) analyses, and the bacterial load is correlated with advanced Dukes stage, microsatellite instability (MSI), CpG island methylator phenotype (CIMP), and adverse prognosis. Strain typing experiments have demonstrated that the oral cavity is the source, with hematogenous translocation and subsequent microbial seeding enabled by transient bacteremia.^26^ A study, Komiya et al.^27^ demonstrated an association between oral and tumor-associated *F. nucleatum* in patients with CRC using arbitrarily primed PCR (AP-PCR). The study revealed the existence of *F. nucleatum* in 100% (14/14) of saliva samples and 57.1% (8/14) of CRC tissues, with 75% (6/8) of individuals positive at both sites, indicating a possible translocation of oral *F. nucleatum* to CRC tissues.^27^ Subsequent colonization of *F. nucleatum* tumor tissues is mediated by glycan‒lectin interactions, with aberrant expression of truncated O-linked glycans, such as Gal-GalNAc, by colorectal adenocarcinomas, which are specifically recognized by the fusobacterial adhesin Fap2, enabling high-affinity adhesion and selective tumor epithelial colonization.^28^

Colonization of the tumor microenvironment facilitates *F. nucleatum* to orchestrate oncogenesis by multiple convergent mechanisms. The surface adhesin FadA interacts with E-cadherin on colonic epithelial cells, enabling clathrin-mediated uptake and destabilization of adherens junctions, stabilizing β-catenin, and activating canonical Wnt signaling. Nuclear β-catenin elevates oncogenic transcriptional targets such as myelocytomatosis (MYC), Cyclin D1 (CCND1), and NF-κB-2, thus supporting proliferation, while Annexin A1 (ANXA1) enables this adhesion process as an essential host cofactor.^29^ Simultaneously, *F. nucleatum* activates the TLR4-MyD88-NF-κB signaling pathway, leading to increased expression of the oncogenic microRNA (oncomiR) miR-21, which inhibits the tumor-suppressor Ras p21 protein activator 1 (RASA1) and activates the RAS Sarcoma-MAPK (RAS-MAPK) pathway, promoting cell invasion and survival. Tumors enriched with *F. nucleatum* have high levels of miR-21 expression, which is correlated with disease stage and low overall survival. These interactions depict a split FadA-E-cadherin and TLR4-MyD88-NF-κB signaling axis involved in the support of *F. nucleatum*-induced colorectal carcinogenesis.^30-32^ In a study by Rubinstein et al.^33^, the oncogenic role of *F. nucleatum* was examined in CRC cell lines (HCT116 and SW480), mouse xenograft models, and clinical tumor samples. The study revealed that *F. nucleatum* has been found to promote tumorigenesis through adhesin FadA binding to E-cadherin in epithelial cells and activating the β-catenin pathway. This results in enhanced oncogenic transcription and cellular proliferation, and inhibition of FadA significantly decreased such effects. Notably, inhibition of FadA using blocking peptides and FadA-deficient mutants blocked E-cadherin binding and β-catenin activation, significantly reduced tumor cell proliferation.^33^

*F. nucleatum* also impacts chemoresistance in CRC through the modulation of host signaling and apoptosis pathways. The activation of the TLR4-MyD88-NF-κB pathway inhibits miR-18a and miR-4802, resulting in the upregulation of the autophagy regulators Unc-51-like kinase 1 (ULK1) and anti-thymocyte globulin 7 (ATG7), which diverts cancer cells from apoptosis to cytoprotective autophagy and resistance to 5-fluorouracil (5-FU) and oxaliplatin.^34^ Simultaneously, the activation of *F. nucleatum* causes the upregulation of baculoviral IAP repeat containing 3 (BIRC3), an apoptosis inhibitor protein that inhibits caspase activation and initiates survival after treatment. Furthermore, TLR4-induced Anoctamin 1 (ANO1) induction reinforces pro-survival and anti-apoptotic signaling, diminishing chemotherapeutic impact.^35^ ^,^ ^36^ Concertedly, these pathways illustrate a TLR4-mediated autophagy and apoptosis-resistance network through which *F. nucleatum* induces therapeutic tolerance and tumor recurrence in CRC. In a study,^34^ the involvement of *F. nucleatum* in chemoresistance was evaluated in CRC cell lines (HCT116 and HT29) and mouse xenograft models, in which *F. nucleatum*induced resistance to 5-FU and oxaliplatin through activation of TLR4-MyD88-NF-κB signaling pathway, leading to downregulation of miR-18a and miR-4802 and upregulation of ULK1 and ATG7. Notably, siRNA-mediated knockdown of TLR4/MyD88 and restoration of miR-18a and miR-4802 expression reversed autophagy activation and restored chemosensitivity, confirming the pathway-specific role of *F. nucleatum* in mediating chemoresistance.^34^

*F. nucleatum* also evades host anti-tumor immunity by engaging in various immune checkpoint-mimicking interactions. The outer membrane adhesin Fap2 has a dual functional domain: the lectin domain binds to tumor-associated Gal-GalNAc glycans, allowing for tumor niche retention, and its non-lectin domain acts directly on the inhibitory receptor TIGIT on natural killer (NK) cells and cytotoxic T lymphocytes to suppress IFN-*γ* release and cytolytic function.^16^ Moreover, CEACAM binding protein of Fusobacterium (CbpF), an outer membrane protein of *F. nucleatum*, interacts with carcinoembryonic antigen-related cell adhesion molecule 1 (CEACAM1) to initiate inhibitory signals that further inhibit lymphocyte-driven killing of tumor cells.^37^ Collectively, these interactions replicate immune checkpoint signaling, which inhibits anti-tumor effector responses and the accumulation of immunosuppressive cell populations in the tumor microenvironment. Such a network of *F. nucleatum*-driven immune evasion drives tumor proliferation and persistence in the face of host immune control.^38^ In a study,^16^ the role of *F. nucleatum* in immune evasion was investigated using primary human NK cells, human Epstein–Barr virus (EBV)-transformed 721.221 cells, CRC cell line RKO, erythroleukemia cell line K562, mouse thymoma BW cells, and NK tumor cell line YTS ECO, which revealed that *F. nucleatum* suppressed immune-mediated cytotoxicity through its adhesin Fap2 by binding to the TIGIT receptor. Notably, Fap2-deficient mutants failed to inhibit immune cell activity, proving its essential role in reducing IFN-*γ* production and cytolytic function.^16^

**Figure 3.**
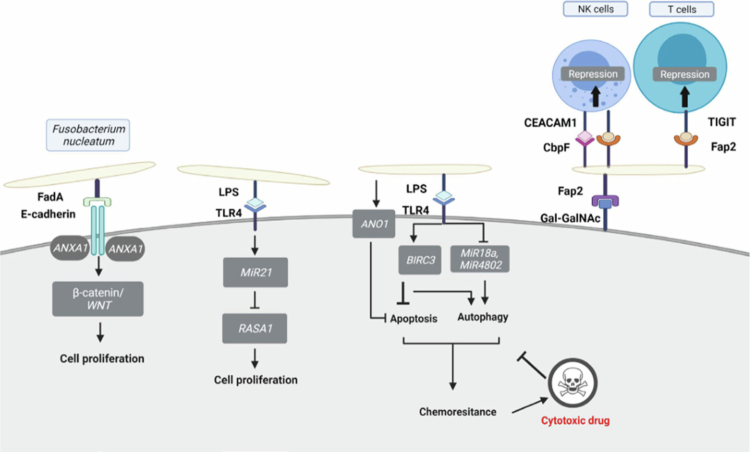
Schematic diagram of various mechanisms employed by *F. nucleatum* to promote CRC. The figure demonstrates the molecular pathways through which *F. nucleatum* contributes to CRC progression. Adhesin FadA binds to E-cadherin on the surface of tumor cells, resulting in the activation of β-catenin/Wnt signaling pathway, which enhances tumor cell proliferation. However, the effect of LPS on the cell surface activates TLR4 through MyD88 pathway, which increases miR-21 expression and downregulates RASA1 expression, thereby promoting tumor cell proliferation. *F. nucleatum* causes chemoresistance by activating TLR4 signaling through MyD88 pathway, which downregulates miR-18a and miR-4802, leading to a shift from apoptosis to autophagy. Additionally, *F. nucleatum* suppresses the host immune response by interacting with the adhesin Fap2 and immune checkpoint receptors such as TIGIT on the surface of T cells and CEACAM1 on the surface of B cells (image adapted from ref.^14^).

The cumulative evidence indicates that *F. nucleatum* is the crucial microbial player in the initiation and progression of CRC, having the ability to affect tumor biology, manipulate the immune environment, and impact clinical outcomes (Figure 3). Multiple studies have provided evidence of the involvement of *F. nucleatum* in CRC progression, variants in experimental approaches and target pathways emphasize the need for standardized approaches. Although, *F. nucleatum* has been extensively investigated in relation to CRC, recent studies indicate that its oncogenic role extends to other types of cancer, including esophageal, gastric, or pancreatic cancer. Mechanistically, similar pathways observed in CRC, such as adhesion-mediated invasion, activation of inflammatory signaling, and modulation of the tumor immune microenvironment, are believed to contribute to tumorigenesis in these cancers. However, the mechanistic and experimental evidence in these malignancies remains limited.^39^

### Adverse pregnancy outcomes

#### Preterm birth (PTB)

*F. nucleatum* has been connected with PTB through hematogenous spread from the oral ecosystem. Periodontal inflammation-mediated transient bacteremia allows the organism to cross the placenta and invade the endothelial tissue.^40^ In a study by Lima et al.^41^ the role of *F. nucleatum* in PTB was evaluated in 120 pregnant women (40 PTB cases and 80 full-term controls) using qPCR analysis of subgingival biofilm samples. The study revealed a significantly higher detection frequency of *F. nucleatum* in women who experienced PTB (35%; 14/40 patients) compared with those who delivered at term (11.2%; 9/80 patients), indicating a potential association between increased maternal oral carriage of *F. nucleatum* and PTB.^41^ The adhesin FadA on *F. nucleatum* interacts with vascular endothelial (VE)–cadherin, disrupting adherens junctions and enhancing endothelial permeability, which promotes bacterial translocation into the decidua and chorionic membranes.^11^ After localization, *F. nucleatum* activates the TLR4-MyD88-NF-κB signaling pathway in trophoblasts and immune cells, resulting in the upregulation of IL-1β, IL-6, TNF-α, and prostaglandin E_2_ (PGE2). These pro-inflammatory mediators induce uterine contractility in uterine tissues and remodeling of the cervix, which eventually leads to premature labor and delivery.^42^

Histopathological analysis of the infected placenta indicates neutrophil infiltration, villous edema, and focal necrosis consistent with infection-mediated chorioamnionitis.^43^ In a study,^44^ the role of *F. nucleatum* in PTB was investigated using a pregnant mouse model, which revealed that intravenous administration of *F. nucleatum* resulted in placental colonization and fetal demise, indicating hematogenous translocation from the oral cavity to the placenta. This resulted in activation of TLR4-MYD88-NF-κB signaling pathway, leading to increased expression of pro-inflammatory cytokines (IL-6 and TNF-α), thereby inducing intrauterine inflammation and preterm delivery. Notably, TLR4 knockout mice (TLR4^−^/^−^) showed significantly reduced placental inflammation and improved pregnancy outcomes.^44^

OMVs and LPS derived from *F. nucleatum* also reveal interference with the integrity of the trophoblastic tight junction, thereby increasing permeability and inflammatory signaling.^22^ Collectively, these evidences confirm that *F. nucleatum* affects placental integrity by FadA-mediated endothelial invasion and TLR4-mediated inflammation, providing a mechanistic link between maternal oral infection and PTB.

#### Chorioamnionitis (CA)

CA, an acute inflammation of the chorion and amnion, has been identified as one of the earliest and well-defined intrauterine infections with *F. nucleatum*. They can colonize the placental–fetal interface through hematogenous dissemination from the maternal oral cavity and induce an inflammatory response. In a study,^45^ the association of *F. nucleatum* with the infection-related CA was investigated in a preterm case of 23 weeks of gestation through microbiological and histopathological examinations, which revealed *F. nucleatum* in fetal lung tissue with severe necrotizing CA.^45^ Subsequently, they stimulate pattern recognition receptors such as Toll-like receptor 2 (TLR2) and TLR4 on trophoblasts and decidual macrophages. This activation promotes NF-κB-mediated transcription of pro-inflammatory cytokines such as IL-1β, IL-6, IL-8, and TNF-α, and the migration of neutrophils and monocytes into the chorioamniotic membranes.^46^ In a study,^47^ Liu et al. demonstrated that *F. nucleatum* induces placental inflammation through activation of TLR4-MyD88-NF-κB pathway in an *in vivo* pregnant mouse model, resulting in increased expression of pro-inflammatory cytokines such as IL-6 and TNF-α.^47^

Elastases, matrix metalloproteinases (MMP-8 and MMP-9), and reactive oxygen species (ROS) are released by invading leukocytes, disrupting components of the extracellular matrix and deteriorating the structural integrity of the fetal membranes, resulting in premature rupture of membranes (PROM), facilitating ascending infection and enhancing placental inflammation.^48^ Histopathological analysis of infected tissues shows dense neutrophilic influx, epithelial detachment, villous necrosis, and edema, characteristics of acute microbial CA.^49^

Collectively, these analyses indicate that *F. nucleatum* is a potent immunostimulatory pathogen in the amniotic environment, and TLR-mediated cytokine cascades and protease activity form the mechanistic platform for *F. nucleatum*-induced CA.

#### Stillbirth and fetal death

*F. nucleatum* has been increasingly implicated as a causative agent for stillbirth and intrauterine fetal death, primarily through hematogenous translocation and colonization of the placenta. Clinical and experimental findings have shown that they can breach the maternal–fetal barrier after bacteremia and selectively accumulate in the placenta, resulting in acute inflammatory damage and placental impairment.^42^ ^,^ ^45^ In a study,^43^ the association between oral and intrauterine *F. nucleatum* was investigated in a term stillborn case (39 weeks + 5 d) using 16S rRNA gene sequencing, which revealed the existence of *F. nucleatum* in placental and fetal tissues, with genetically identical strains identified in maternal subgingiva.^43^ Mechanistically, this tissue tropism is mediated by the adhesin Fap2, a galactose-binding lectin that targets Galβ1-3GalNAc (Thomsen–Friedenreich antigen) and associated T/Tn glycans expressed in high density on trophoblast surfaces. This glycan‒lectin interaction allows for high-affinity adhesion, immune evasion, and chronic bacterial colonization in the chorionic villi.^45^

Adhesion of *F. nucleatum* triggers TLR4-mediated inflammatory signaling, resulting in neutrophil and macrophage recruitment, IL-6, TNF-α, and chemokine local production, and thrombosis of microvessels and tissue necrosis. These pathophysiologic changes compromise maternal–fetal oxygen exchange, resulting in hypoxia, placental infarction, and fetal death.^50^ In a study,^47^ the role of *F. nucleatum* in fetal death was investigated using pregnant mouse models, including TLR4-deficient (C57BL/6 TLR4^−^/^−^ and C3H/HeJ) and TLR2-deficient mice, which revealed that *F. nucleatum* activated both TLR2 and TLR4 *in vitro*; however, *in vivo* fetal death was significantly reduced only in TLR4-deficient mice, accompanied by decreased placental inflammation and necrosis, while TLR2 deficiency showed no protective effect. Notably, treatment with a synthetic TLR4 antagonist (TLR4A) significantly decreased fetal death and decidual necrosis without affecting bacterial colonization, thereby demonstrating that TLR4-mediated inflammatory responses are the primary driver of infection-induced fetal loss.^47^

#### Preeclampsia (PE)

Emerging evidence incriminates *F. nucleatum* as a potential microbial contributor to PE, a hypertensive pregnancy disorder characterized by systemic endothelial dysfunction and placental ischemia. Periodontal infection creates a chronic reservoir for *F. nucleatum* and enables recurrent low-grade bacteremia that exposes maternal vascular endothelium and placental tissues to their components.^22^ ^,^ ^51^ In a study,^52^ the placental microbiomes of preeclamptic pregnancies (*n* = 55) and normotensive controls (*n* = 55) were analyzed using 16S rRNA gene sequencing, which revealed the existence of *F. nucleatum* DNA in 12.7% (7/55) of preeclamptic placentas compared to 0% (0/55) in controls, suggesting a potential link in the pathogenesis of PE.^52^ The LPS and OMVs activate TLR4 signaling in endothelial and trophoblastic cells, which triggers NF-κB-induced inflammatory cascades and the upregulation of IL-6, TNF-α, and endothelin-1. These mediators induce oxidative stress, vasoconstriction, and reduced nitric oxide bioavailability, thus diminishing vascular relaxation.^53^ In a study, Liu et al.^47^ demonstrated that *F. nucleatum* can colonize placental tissues and induce inflammatory responses through activation of the TLR4-MyD88-NF-κB signaling pathway in an *in vivo* pregnant mouse model, leading to increased expression of pro-inflammatory cytokines such as IL-6 and TNF-α. These inflammatory and endothelial modifications disrupt placental function and are mechanistically relevant to key pathological features associated with PE.^47^

Chronic exposure of the placenta to *F. nucleatum* disrupts trophoblast differentiation and angiogenesis, causing an imbalance between proangiogenic factors such as vascular endothelial growth factor (VEGF), placental growth factor (PIGF), and antiangiogenic factors (soluble fms-like tyrosine kinase-1, soluble endoglin). This antiangiogenic shift impairs spiral artery remodeling, with resultant placental hypoperfusion and ischemia, which is central to PE pathogenesis.^54^ Histological assessment of infected placentas shows villous fibrosis, endothelial swelling, and inflammatory infiltration, consistent with vascular stress and immune activation.^55^

These evidences collectively indicate that *F. nucleatum* acts as an inflammatory cofactor for the exacerbation of placental dysfunction and hypertension during pregnancy through LPS-mediated endothelial activation and angiogenic dysregulation, thus linking maternal oral dysbiosis to vascular complications during gestation.^22^

#### Low birth weight (LBW)

Chronic infection of the placenta with *F. nucleatum* has been strongly linked to LBW, reflecting intrauterine growth restriction due to dysfunction of the placenta. Hematogenous dissemination from the oral cavity allows continued colonisation at the placenta interface where LPS and OMVs lead to continuous TLR4-mediated inflammatory pathway activation.^55^ Sustained activation of NF-kB signaling causes a pronounced release of interleukin 6 (IL-6), tumor necrosis factor alpha (TNF-alpha), and reactive oxygen species (ROS), which generate a pro-inflammatory and oxidative microenvironment that impairs trophoblast viability.^4^ In a study, Rubinstein et al.^33^ demonstrated that *F. nucleatum* invades host tissues through FadA, which binds to vascular endothelial cadherin (VE-cadherin) and disrupts endothelial integrity. This results in bacterial translocation into placental tissues and facilitates localized inflammation. Furthermore, it disrupts vascular function and nutrient transfer, leading to fetal growth restriction and LBW.^33^

The capacity of the placental barrier for efficient nutrient and gas exchange is compromised with prolonged infection, as it suppresses trophoblast proliferation, differentiation and syncytialization. Increased levels of TNF-α and IL-6 down-regulate insulin-like growth factor-1 (IGF-1) and the expression of nutrient transporters, including SLC38A2 and GLUT-1, which has the consequence of reducing the fetal supply of nutrients.^56^ The *F. nucleatum* induced inflammatory environment disturbed the angiogenic balance by the inhibition of VEGF expression and the formation of a capillary network in the villous tissues.^54^

**Figure 4.**
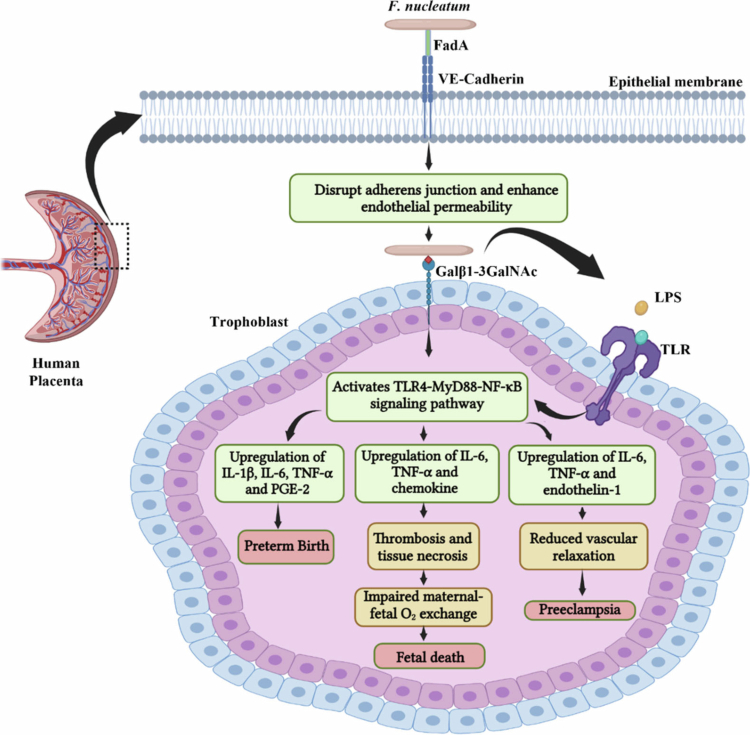
Mechanistic illustration of the role of *F. nucleatum* in adverse pregnancy outcomes: The adhesin FadA on *F. nucleatum* interacts with VE-cadherin on endothelial cells, disrupting adherens junctions and increasing endothelial permeability, thereby facilitating the bacterial translocation into placental tissues. Furthermore, Fap2 interaction with Galβ1-3GalNAc enhances adhesion to trophoblasts and results in the activation of TLR4-MyD88-NF-κB signaling pathway and the upregulation of proinflammatory cytokines such as IL-1β, IL-6, TNF-α, and chemokines. These inflammatory responses promote uterine contractility, leading to PTB, while also inducing thrombosis, tissue necrosis, and impaired maternal–fetal oxygen exchange, contributing to fetal death. In addition, LPS-mediated TLR4 activation results in increased IL-6, TNF-α, and endothelin-1 levels, causing oxidative stress, vasoconstriction, and reduced nitric oxide bioavailability, thereby leading to endothelial dysfunction and PE.

These mechanisms are supported by epidemiological evidence showing a strong positive relationship between maternal periodontal disease and/or a positive relationship with the incidence of LBW in babies. Collectively, these data support the role of *F. nucleatum* in chorionic placental inflammation, angiogenic impairment and metabolic dys-regulation in setting the restrictive limits of fetal growth, thus providing a direct microbiological link between dysbiosis in the mother's oral cavity and poor neonatal development.^55^ Although, clinical and experimental investigations support the involvement of *F. nucleatum* in adverse pregnancy outcomes, differences in study design and reliance on simplified models indicate the need for physiologically relevant and longitudinal investigations (Figure 4).

### Cardiovascular diseases

#### Atherosclerosis

Molecular and clinical evidence implicates *F. nucleatum* as a microbial contributor to atherosclerosis, which is a chronic inflammatory disease of the arterial wall. In a study,^57^ the existence of *F. nucleatum* was evaluated using qPCR analysis of oral samples from subjects with atherosclerotic cardiovascular diseases (*n* = 40), familial hypercholesterolemia (*n* = 26), and healthy controls (*n* = 31), in which *F. nucleatum* was detected across all groups.^57^ Experimental studies on ApoE^−^/^−^ mouse models have demonstrated that the oral administration of *F. nucleatum* leads to a significant increase in atherosclerotic plaque formation.^10^ Periodontal bacteremia enables *F. nucleatum* to gain access into the systemic circulation, by which they bind to the vascular endothelium.^56^ The adhesin FadA interacts with VE-cadherin, disrupting endothelial junctions and permitting microbial invasion through the subendothelial layer.^11^ Internalized *F. nucleatum* triggers TLR2 and TLR4 on endothelial and immune cells, engaging NF-κB-induced transcription of IL-6, TNF-α, IL-1β, and monocyte chemoattractant protein-1 (MCP-1). These cytokines facilitate the recruitment of monocytes and their differentiation into foam cells, a key event in early plaque formation. In a study by Zhou et al.^10^ the role of *F. nucleatum* in atherosclerosis was investigated using ApoE^-/-^ mouse models and *in vitro* macrophage systems, which revealed that the oral administration of *F. nucleatum s*ignificantly enhances plaque formation through TLR2/TLR4-NF-κB activation, leading to cytokine upregulation, foam cell formation, and lipid accumulation.^10^

It has been shown that LPS and OMVs derived from *F. nucleatum* increase oxidative stress and increase expression of the adhesion molecules intercellular adhesion molecule-1 (ICAM-1) and vascular cell adhesion molecule-1 (VCAM-1) to facilitate leukocyte adhesion and transmigration into the intima.^58^ *F. nucleatum* facilitates lipid accumulation in atherosclerotic lesions, which is responsible for the formation of the necrotic core development and plaque instability.^10^ Experimental models have shown that chronic exposure to LPS increases the progression of atherosclerotic plaque with endothelial dysfunction.^59^ Moreover, *F. nucleatum* DNA has been found in human carotid and coronary atheromas by 16S rRNA sequencing and in situ hybridization, confirming their direct role in vascular pathology.^60^

Collectively, these findings identified *F. nucleatum* as a vascular pathobiont that promotes the development of atherosclerosis through vascular endothelial invasion, inflammation, and lipid dysregulation and is thus linked to chronic oral infection, vascular inflammation, and the progression of vascular plaque. Despite growing evidence supporting the role of *F. nucleatum* in atherosclerosis, variations in experimental models and limited strain-specific analysis highlight the need for more standardized and mechanistically robust studies.

#### Infective endocarditis (IE)

*F. nucleatum* is an oral commensal that has been recognized as a clinically significant pathogen in IE, a disease that features microbial colonization of the endocardial surface and cardiac valves. In a study, Ioannou et al.^61^ evaluated the association between *F. nucleatum* and IE, which revealed the presence of *F. nucleatum* in 42.9% (9/21) of IE patients, with a frequent association with 77.8% of oral risk factors, suggesting the oral origin of *F. nucleatum*. They have entered the bloodstream as a result of periodontal inflammation, dental manipulation, or mucosal disruption, thereby inducing transient bacteremia.^61^ Once circulating, *F. nucleatum* uses their adhesins and outer membrane proteins consisting of FadA, RadD, and FomA to attach to fibrin‒platelet aggregates and expose endothelial sites on damaged cardiac valves. This attachment helps to form a vegetative biofilm, which provides them a protection against host immune clearance and penetration of antibiotics.^62^

*F. nucleatum* stimulates TLR2/TLR4 signaling in endothelial cells, leading to the synthesis of pro-inflammatory cytokines (IL-1β, IL-6, and TNF-α) and the upregulation of tissue factors involved in the coagulation and development of vegetative structures.^63^ Endothelial cytotoxicity, platelet aggregation, and thrombus maturation are also regulated by the release of SCFA and OMVs.^64^ Histopathological examinations also showed the presence of *F. nucleatum* DNA and live organisms from resected cardiac valves and vegetations, which supported their role in the pathogenesis of IE.^65^ In a study,^63^ the role of *F. nucleatum* in IE was investigated using *in vitro* human endothelial cell models, which revealed that *F. nucleatum* activates TLR2/TLR4-mediated signaling pathways, leading to significant upregulation of pro-inflammatory cytokines such as IL-1β, IL-6, and TNF-α and tissue factor expression, promoting coagulation. Notably, inhibition of TLR signaling significantly reduced cytokine production and endothelial activation, indicating an essential role in inflammation-mediated vegetation formation.^63^

Clinically, IE due to *F. nucleatum* is usually a subacute infection with prolonged fever, embolisms, and systemic inflammation, exacerbating pre-existing periodontal disease. Long-term bactericidal antibiotic treatment is essential due to biofilm-associated tolerance and innate resistance to β-lactams.^61^ Collectively, these results delineate *F. nucleatum* as a rare but mechanistically significant cause of IE that results from oral-derived bacteremia and the development of biofilms on the cardiac endothelium. Although clinical and molecular evidence supports the involvement of *F. nucleatum* in IE, limited experimental validation and small sample sizes indicate the need for further investigations.

### Neurogenerative disorders

#### Alzheimer’s disease (AD)

Accumulating evidence outlined the involvement of *F. nucleatum* in the pathogenesis of AD through mechanisms focused on chronic neuroinflammation and dysfunction of the blood‒brain barrier (BBB).^12^ Periodontal infection is one route through which *F. nucleatum* may enter systemic circulation; therefore, their LPS, OMVs, and SCFAs reach the central nervous system (CNS) through a compromised BBB.^66^ These components have been identified in post-mortem AD brain tissues, hence supporting hematogenous spread. LPS activates TLR2 and TLR4 on microglia and astrocytes, thereby initiating NF-κB-dependent transcription of IL-1β, IL-6, TNF-α, and ROS.^67^

**Figure 5.**
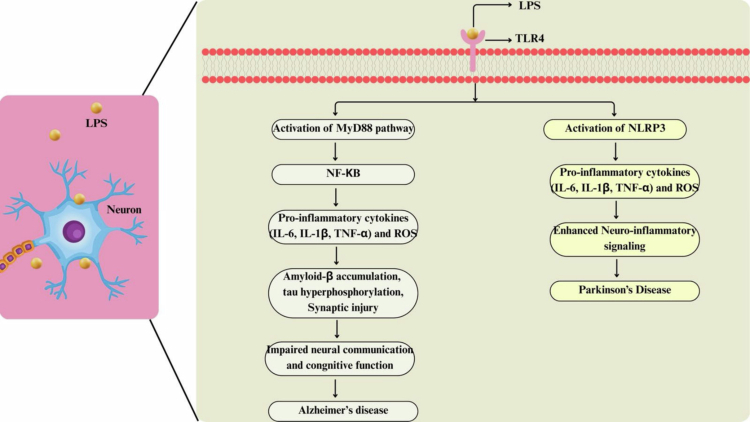
Mechanistic illustration of the involvement of *F. nucleatum* in neurodegenerative diseases: LPS of *F. nucleatum* activates the TLR4 signaling pathway in neuronal and glial cells, initiating the MyD88-NF-κB signaling cascade and the NLRP3 inflammasome activation pathway. This leads to increased production of ROS and proinflammatory cytokines (IL-6, IL-1β, and TNF-α). The resulting neuroinflammatory environment promotes the accumulation of amyloid-β plaques and tau protein hyperphosphorylation, leading to synaptic dysfunction, impaired neuronal communication, and cognitive function, thereby contributing to AD. Concurrently, the NLRP3-mediated inflammatory response activates the neuroinflammatory reactions and contributes to the pathophysiology of PD.

Persistent inflammatory signaling promotes amyloid-β accumulation, tau hyperphosphorylation, and synaptic injury, collectively impairing neuronal communication and cognitive function (Figure 5).^42^ SCFAs produced by *F. nucleatum* affect the metabolism of neurons and the homeostasis of the mitochondria, thus contributing to neurodegeneration.^68^ In a study, Yan et al.^67^ evaluated the role of *F. nucleatum* in AD using AD-like rodent model, where oral infection with *F. nucleatum* resulted in a significant increase in the deposition of β-amyloid and tau protein hyperphosphorylation (*p* < 0.05) by immunohistochemical and molecular analysis. This study also revealed a significant increase in pro-inflammatory cytokines, demonstrating that microglial activation by TLR-mediated signaling. Moreover, it demonstrated the existence of *F. nucleatum* DNA in brain tissues by PCR, indicating hematogenous translocation. Further, the study revealed significant impairments in spatial memory in infected mice, proving the potential role of *F. nucleatum* in neuroinflammation and neurodegeneration.^67^

Collectively, *F. nucleatum* functions as a pro-inflammatory microbial cofactor in the pathophysiology of AD, connecting chronic oral dysbiosis to neuroinflammatory and amyloidogenic processes that exacerbate cognitive decline.

#### Parkinson’s disease (PD)

Recent research links *F. nucleatum* infection to PD primarily through gut‒brain axis dysregulation and chronic systemic inflammation. Oral and intestinal colonization by *F. nucleatum* disturb the gut microbiome, increasing intestinal permeability and thereby allowing the translocation of bacterial LPS and metabolites into the circulation.^69^ These microbial compounds stimulate TLR4 and the NOD-like receptor (NLRP3) inflammasome to trigger IL-1β and TNF-α secretion and enhance neuroinflammatory signaling (Figure 5).^70^

Systemic inflammation favors α-synuclein misfolding within enteric neurons and microglial activation in the substantia nigra, promoting dopaminergic neuronal degeneration. *F. nucleatum*-derived SCFAs, mainly butyrate and propionate, affect vagal and immune signaling pathways, influencing neuro-glial communication.^70^ In a study,^69^ Chronic oral infection of C57BL/6 rodent models with *F. nucleatum* for 6 weeks led to significant cognitive dysfunction accompanied by significant neuronal degradation in the hippocampus and cortex. This resulted in elevated LPS levels and microglial cell activation, leading to the release of pro-inflammatory cytokines (IL-1β, TNF-α, and IL-6) through TLR-mediated pathways.^69^

The ability of the *F. nucleatum* to induce oxidative stress, impair tight junctions in the gut epithelium, and promote systemic endotoxemia and neuroinflammation. Collectively, these results substantiate a mechanistic model in which *F. nucleatum*, through disruption of the gut microbiota, LPS-induced inflammation, and mitochondrial stress, contributes to the pathogenesis of PD by reinforcing the bidirectional inflammatory axis between the gut and brain.^70^ While multiple studies support the involvement of *F. nucleatum* in neuroinflammation, current evidence remains largely indirect, indicating the need for direct mechanistic and longitudinal studies.

#### Diabetes mellitus (DM)

*F. nucleatum* has been increasingly implicated in the development and progression of DM through mechanisms that include chronic inflammation, insulin resistance, and metabolic dysregulation.^71^ Chronic periodontal infection with *F. nucleatum* causes systemic low-grade inflammation, with increased levels of IL-1β, IL-6, and TNF-α, which are known to interfere with insulin receptor substrate (IRS) signaling and impair glucose uptake in peripheral tissues.^72^ Their LPS and OMVs activate the TLR4 and NF-κB pathways in adipocytes, hepatocytes, and pancreatic beta (β)-cells and thus drive the transcription of inflammatory mediators and stress kinases, including c-Jun *N*-terminal kinase (JNK) and p38 MAPK, which antagonize insulin signaling cascades.^71^

Cytokines stimulated by *F. nucleatum* result in β-cell dysfunction and apoptosis with a consequent decrease in the capacity to secrete insulin.^72^ The metabolic products, especially SCFAs and formate, act on hepatic gluconeogenesis, oxidative stress, and further contribute to hyperglycemia.^73^ Clinical studies have documented high serum antibody levels and the identification of DNA in patients who have poorly controlled diabetes, supporting a systemic microbial association^74^ Moreover, hyperglycemia favors oral dysbiosis and significantly increases the proliferation of *F. nucleatum*, establishing a mutual relationship between infection and metabolic imbalance.^74^ In a study,^75^ the role of *F. nucleatum* in type 2 DM was investigated through single-cell RNA sequencing of human pancreatic islet cells and MIN6 cells (a mouse pancreatic β-cell line), which revealed that *F. nucleatum* is correlated with a increased transdifferentiation markers and a decreased proportion of functional β-cells. It demonstrates that *F. nucleatum* activates the NF-κB signaling pathway and results in β-cell identity. The impairment of β-cell function contributes to defective insulin secretion and the progression of diabetes.^75^

Collectively, these findings highlight *F. nucleatum* as a pro-inflammatory and metabolic disrupter and serve as a link between periodontal infection and the systemic inflammatory environment in the body, which leads to insulin resistance and glycemic dysregulation and supports the role of microbial contribution to the pathophysiology of DM.

### Therapeutic implications

The understanding of *F. nucleatum* as a systemic pathogen requires targeted therapeutic measures beyond conventional antibiotic therapy. Current and emerging interventions are based on neutralizing key virulence determinants, which are aimed at reestablishing the microbial homeostasis.^76^ Anti-adhesin strategies that can either target critical adhesin–receptor interactions, such as FadA–E-cadherin and Fap2–GalNAc with small-molecule inhibitors or glycan mimetics, prevent epithelial invasion and colonization of metastases.^77^ Correspondingly, OMV integrated approaches, such as nanobody-based neutralization and protease inhibitors, which have been developed to inhibit the delivery of pro-inflammatory and immunosuppressive vesicular cargo.^78^ ^,^ ^79^

Precision modulation of the microbiome using the administration of probiotics, prebiotics, and engineered commensals is a feasible non-antibiotic strategy for the competitive suppression of *F. nucleatum* within oral and intestinal biofilms.^80^ Specialized methods such as bacteriophage treatment and CRISPR-Cas-guided editing selectively remove virulent strains without affecting the diversity of the commensals.^81^ Host-directed therapeutics by blocking TLR4-NF-κB signaling, modulating anti-inflammatory miRNA, and immune checkpoint antagonism have the potential to mitigate the immune evasion and chronic inflammation mediated by *F. nucleatum* infection.^77^ Emerging preclinical data demonstrate that eliminating *F. nucleatum* in combination with chemotherapy or immune checkpoint blockade may enhance drug sensitivity and tumor regression.^82^

### Future perspectives

Although there are increasing number of studies reporting the association of *F. nucleatum* with various systemic diseases, the current researches are still limited by a lack of longitudinal clinical trials, insufficient integration of strain-specific virulence mechanisms, and reliance on fragmented multi-omics approaches, which collectively make it difficult for translation into clinical applications. These gaps highlight the need for targeted strategies that can directly interfere with bacterial‒host interactions. Future studies must utilize systems biology and multi-omics to understand host–pathogen dynamics at single-cell resolution in order to translate from descriptive correlation to mechanistic intervention. Determining integrative metagenomics, transcriptomics, proteomics, and metabolomics will be key to defining strain-specific virulence markers, metabolic cross-talk, and immune interactions. Artificial intelligence and machine learning models can rapidly identify novel druggable targets and forecast host susceptibility based on microbial and immunogenomic profiles.

Particularly, adhesion-mediated interactions involving FadA/E-cadherin or Fap2/host cell receptor targeting have been identified as therapeutic opportunities. Future studies should focus on recombinant peptide-based therapies that could be used for competitive inhibition of *F. nucleatum* adhesion to host cells, thereby preventing colonization, invasion, and further inflammatory signaling. This might be highly specific with minimal impact on the microbiota.

In addition to these targeted anti-adhesion strategies, broader therapeutic approaches aimed at eliminating or modulating *F. nucleatum* should also be explored. Therefore, translational advances in the development of vaccines based on OMV or adhesins, biofilm-targeting nanotherapeutics, and phage-nanoparticle conjugates are needed to achieve selective clearance of *F. nucleatum* without ecological disruption. Moreover, establishing a system of strain-resolved clinical surveillance and longitudinal human cohorts will allow for risk stratification and monitoring of treatment efficacy. Preclinical validation with organ-on-chip and 3D co-culture models will be more representative of the spatial and metabolic complexity of host‒microbe interactions.

Consequently, a combination of oral health management with precision microbial therapeutics and host-targeted modulation might redefine the clinical paradigm of *F. nucleatum-*associated diseases and transform the once-commensal bacterium into a preventable systemic risk factor.

## Conclusion

*F. nucleatum* has moved beyond its status as an oral commensal to a keystone pathobiont that connects local dysbiosis with systemic pathology. Their distinctive molecular armamentarium, consisting of adhesins (FadA, Fap2, and RadD), outer membrane vesicles, LPS, and SCFAs, orchestrates a multifaceted interplay between microbial communities and host signaling networks.^14^ ^,^ ^42^ ^,^ ^68^ *F. nucleatum* is associated with the pathogenesis of various neoplastic and systemic diseases. It is best studied in the context of CRC, with its role also being recognized in other gastrointestinal malignancies, such as gastric, esophageal, and pancreatic cancer, but mechanistic evidence in these contexts remains comparatively limited. It also contributes to distal pathogenic manifestations, including poor pregnancy outcomes, cardiovascular diseases, neurodegenerative diseases, diabetes mellitus, and periodontal inflammation.^14^ ^,^ ^22^ ^,^ ^42^ ^,^ ^67^

Their ability to modulate innate and adaptive immune responses, to take advantage of the host adhesion entry, and to trigger chronic low-grade inflammation makes *F. nucleatum* the central microbial mediator of systemic diseases. Accumulating evidence indicates their dual role in the initiation and progression of diseases, providing their relevance in diagnostic, prognostic, and therapeutic considerations.^83-85^ Although huge strides have been made in the decipherment of their pathogenic mechanisms, therapeutic translation is still in its infancy. Further interventions are needed to integrate anti-adhesion strategies, microbiome control, and host-directed therapeutics in precision medicine frameworks.^77^ Concurrently, multi-omics and AI-driven analyses will be of critical importance to help identify strain-level virulence markers, predict human disease susceptibility, and personalize interventions.^86^ ^,^ ^87^

## Data Availability

Data sharing is not applicable to this article as no new data were created or analyzed in this study.
